# Association of serum 25-hydroxyvitamin D with urinary albumin-to-creatinine ratio and diabetic retinopathy in hospitalized patients with type 2 diabetes mellitus: a cross-sectional study

**DOI:** 10.1186/s12902-026-02307-w

**Published:** 2026-05-11

**Authors:** Xiang-Yu Chen, Fan-Rui Meng, Yang Zhang, Xiang-Xin Wang, Mei Xue, Wen-Hua Xiao

**Affiliations:** 1 Department of Endocrinology, Peking University Third Hospital Qinhuangdao Hospital, Qinhuangdao, 066000 China; 2 Department of Endocrinology and Metabolism, Peking University Third Hospital, Beijing, China; 3 Department of Endocrinology, Zhongnan Hospital of Wuhan University, Wuhan, 430071 China

**Keywords:** Type 2 diabetes mellitus, 25-hydroxyvitamin D, Urinary albumin-to-creatinine ratio, Diabetic retinopathy, Nonlinear association

## Abstract

**Background:**

Vitamin D deficiency is common in patients with type 2 diabetes mellitus (T2DM), but its association with diabetic microvascular complications remains unclear. We investigated the relationships between serum 25-hydroxyvitamin D [25(OH)D] levels and urinary albumin-to-creatinine ratio (UACR) as well as diabetic retinopathy (DR) in patients with T2DM.

**Methods:**

In total, 518 patients with T2DM were included and classified into vitamin D deficient (< 20 ng/mL) and non-deficient (≥ 20 ng/mL) groups according to serum 25(OH)D levels. Missing covariate data were handled using multiple imputation. Baseline characteristics were compared between groups. The associations of serum 25(OH)D with ln-transformed UACR, UACR ≥ 30 mg/g, and DR were evaluated using multivariable linear and logistic regression models. Subgroup analyses, interaction tests, and restricted cubic spline analyses were performed to examine effect modification and dose-response relationships. Seasonal variation in serum 25(OH)D was additionally assessed in an expanded cohort of 970 patients with T2DM.

**Results:**

Patients with vitamin D deficiency had higher HbA1c, triglycerides, LDL-C, and UACR than those without deficiency. In the expanded seasonal analysis, serum 25(OH)D levels in patients with T2DM showed significant seasonal variation, with the lowest levels in autumn, while no significant differences were observed among spring, summer, and winter. Serum 25(OH)D was inversely associated with ln(UACR) in the multivariable model (adjusted B = − 0.070, 95% CI: −0.106 to − 0.033; *P* < 0.001) and with UACR ≥ 30 mg/g (adjusted OR = 0.933, 95% CI: 0.887–0.981; *P* = 0.006). This association was more evident in younger patients. Restricted cubic spline analysis suggested a non-linear inverse relationship between 25(OH)D and lnUACR. Serum 25(OH)D was also inversely associated with DR risk (adjusted OR = 0.932, 95% CI: 0.877–0.991; *P* = 0.024), although no significant interaction was observed in subgroup analyses.

**Conclusions:**

Lower serum 25(OH)D levels were associated with higher UACR and showed a modest association with DR in hospitalized patients with T2DM, warranting further investigation in prospective studies.

**Clinical trial number:**

Not applicable.

**Supplementary Information:**

The online version contains supplementary material available at 10.1186/s12902-026-02307-w.

## Introduction

Diabetic microvascular complications, particularly diabetic kidney disease (DKD) and diabetic retinopathy (DR), remain major causes of impaired quality of life, excess cardiovascular risk, and premature mortality among patients with type 2 diabetes mellitus (T2DM) [1, 2]. Although glycemic control, blood pressure management, and lipid lowering are central to prevention, many patients still develop or experience progression of microvascular damage despite achieving conventional treatment targets [3]. This residual burden suggests that factors beyond traditional metabolic risk markers may contribute to disease heterogeneity and has been partly attributed to metabolic memory [3].

Vitamin D, assessed by circulating 25-hydroxyvitamin D [25(OH)D], has attracted attention because its effects extend beyond bone metabolism, and deficiency is common [4, 5]. Experimental and clinical studies suggest that vitamin D signaling modulates immune and inflammatory responses, attenuates oxidative stress, preserves endothelial function, and influences the renin–angiotensin–aldosterone system [6–9]. In parallel, epidemiological studies have linked lower 25(OH)D levels to type 2 diabetes risk, insulin resistance, kidney function, DKD-related measures, and DR [10–16]. Ageing-related changes in kidney structure and function, together with reduced vitamin D activity in older adults, provide a biologically plausible basis for effect modification.

However, several important gaps remain. First, most prior studies have assumed a linear exposure–response relationship, without formally examining whether the association differs at lower versus higher 25(OH)D concentrations. Second, interindividual heterogeneity has not been adequately addressed. Vitamin D responsiveness may be altered by age-related physiological changes [17–20], and longer diabetes duration may reflect cumulative metabolic and microvascular burden that could modify observed associations. Direct evidence from a unified analytical framework remains limited.

To address these gaps, we conducted a real-world observational study in hospitalized patients with T2DM, using restricted cubic spline (RCS) models and formal interaction analyses to characterize the association between serum 25(OH)D and microvascular phenotypes. Specifically, we aimed to:

1. Examine potential non-linear relationships between serum 25(OH)D and UACR and DR;
2. Assess whether age and diabetes duration modify these associations.

## Materials and methods

### Study design

This was a single-center, retrospective cross-sectional study conducted in the Department of Endocrinology at our hospital between January 2023 and April 2025. Clinical and laboratory data were extracted from the electronic medical record system.

The study was conducted in accordance with the Declaration of Helsinki and was approved by the Institutional Ethics Committee of Peking University Third Hospital Qinhuangdao Hospital (Approval No. A004; approval date: June 19, 2024). Due to the retrospective design and use of anonymized data, the requirement for informed consent was formally waived by the Ethics Committee.

### Study population and eligibility criteria

Patients hospitalized during the study period were screened. T2DM was diagnosed according to World Health Organization criteria: fasting plasma glucose ≥ 7.0 mmol/L, 2-hour plasma glucose ≥ 11.1 mmol/L during a 75-g oral glucose tolerance test (OGTT), glycated hemoglobin (HbA1c) ≥ 6.5%, or random plasma glucose ≥ 11.1 mmol/L in patients with classic symptoms of hyperglycemia.

Exclusion criteria were vitamin D or calcium supplementation within the preceding 3 months; endocrine disorders affecting calcium–phosphate or vitamin D metabolism; severe acute infection, acute cardiovascular or cerebrovascular events, advanced hepatic or renal dysfunction (eGFR < 30 mL/min/1.73 m²), active malignancy; and pregnancy or lactation.

**Fig. 1:**
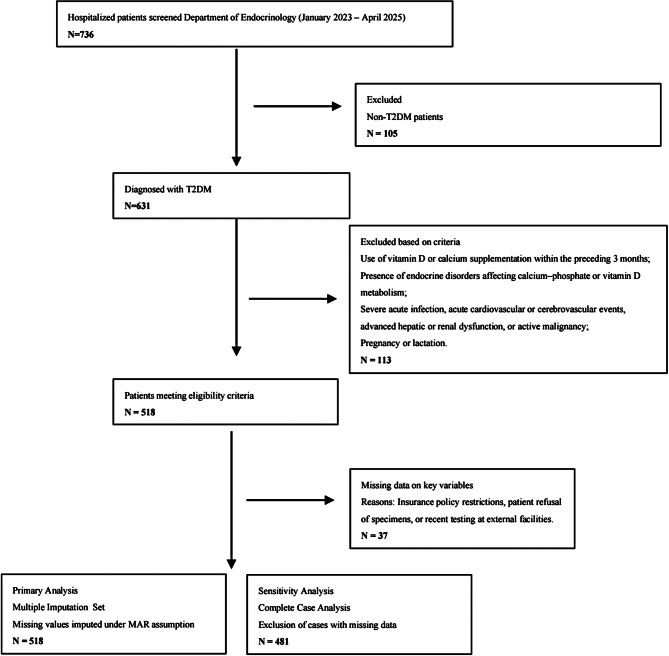
Flowchart of patient selection and exclusion

Of 736 screened patients, 105 were excluded for not meeting the T2DM criteria and 113 were excluded by predefined exclusion criteria, leaving 518 eligible participants. Missing data on key variables were present in 37 participants. Multiple imputation (MI) under the missing at random (MAR) assumption was used for the primary analysis (*N* = 518), and complete-case analysis (CCA; *N* = 481) was performed as a sensitivity analysis(Fig. 1).

A total of 736 hospitalized patients were initially screened, with 518 meeting the eligibility criteria after sequential exclusions. For statistical analysis, the cohort was divided into: (1) the primary analysis set (*N* = 518), utilizing multiple imputation to address missing data (*n* = 37); and (2) the sensitivity analysis set (*N* = 481), consisting of complete cases only.

### Data collection and measurements

Baseline demographic and clinical characteristics, including sex, age, body mass index (BMI), and diabetes duration, were obtained from electronic medical records.

After an overnight fast of at least 8 h, venous blood samples were collected for measurement of serum 25(OH)D, calcium, phosphorus, creatinine, HbA1c, albumin, hemoglobin, triglycerides, and low-density lipoprotein cholesterol. Serum 25(OH)D was measured by chemiluminescence immunoassay under strict quality control. Vitamin D deficiency was defined as serum 25(OH)D < 20 ng/mL (50 nmol/L), consistent with the Endocrine Society clinical practice guidelines [4]. This threshold has been widely used in epidemiological studies and corresponds to the level below which secondary hyperparathyroidism and bone turnover markers may increase.

Medication exposure before admission was defined as continuous use for at least 1 month. Antidiabetic medications were classified as metformin, glucagon-like peptide-1 receptor agonists (GLP-1 RA), sodium-glucose cotransporter 2 inhibitors (SGLT2i), insulin, and other antidiabetic agents. Use of angiotensin-converting enzyme inhibitors/angiotensin receptor blockers (ACEI/ARB) was also recorded.

DR was diagnosed by experienced ophthalmologists according to standardized criteria based on funduscopic examination and fundus photography. Urinary albumin-to-creatinine ratio (UACR) was measured in a morning spot urine sample, and abnormal UACR was defined as UACR ≥ 30 mg/g.

### Statistical analysis

Statistical analyses were performed using SPSS software (version 31.0), R (version 4.5.2) for restricted cubic spline analyses, and GraphPad Prism (version 10.4.2) for graphical presentation. Continuous variables were assessed for normality using the Shapiro–Wilk test and are presented as mean ± SD or median (IQR), as appropriate. Categorical variables are presented as numbers and percentages. Group comparisons were performed using independent-samples t-tests, Mann–Whitney U tests, Welch’s t-tests, or chi-square tests, as appropriate.

Multiple imputation under the missing at random assumption was used as the primary approach to handle missing data (*n* = 37). Twenty imputed datasets were generated, and pooled estimates were obtained using Rubin’s rules. Complete-case analysis was performed as a sensitivity analysis. Because UACR was skewed, it was natural log-transformed [ln(UACR)] before analysis. Multivariable linear regression was used to assess the association between serum 25(OH)D and ln(UACR), and multivariable logistic regression was used for UACR ≥ 30 mg/g and DR. Unless otherwise specified, all models were adjusted for age, sex, BMI, duration of T2DM, HbA1c, eGFR, hypertension, and medication use (metformin, SGLT2 inhibitors, insulin, and ACEI/ARB).

Interaction terms were used to assess effect modification by age, duration of T2DM, sex, ACEI/ARB use, and eGFR. Age was dichotomized at 59 years, approximately the median of the study population, and duration of T2DM at 7 years, the median value of the cohort, with additional cut-offs used in sensitivity analyses. Serum 25(OH)D, age, duration of T2DM, and eGFR were mean-centered before inclusion of interaction terms. Restricted cubic spline analyses with four knots at the 5th, 35th, 65th, and 95th percentiles of serum 25(OH)D (11.87, 16.18, 19.01, and 26.86 ng/mL, respectively) were used to examine dose–response relationships. A reference value of 20 ng/mL was set for estimation. Overall and nonlinear associations were tested using Wald tests.

Seasonal variation in serum 25(OH)D was descriptively evaluated in an expanded cohort of 970 patients with T2DM, which included the primary study population. Participants were categorized into winter, spring, summer, and autumn according to the date of blood sampling. One-way ANOVA was used to compare serum 25(OH)D levels across seasons. This analysis was conducted for descriptive purposes only and was not included in the primary regression models.

## Results

### Baseline characteristics of the study population

A total of 518 patients with T2DM were included in the study and categorized into vitamin D deficient (< 20 ng/mL, *n* = 372) and non-deficient (≥ 20 ng/mL, *n* = 146) groups based on serum 25(OH)D levels (Table 1). The baseline characteristics of the complete-case dataset are presented in Supplementary Table S1.

In terms of demographic characteristics, patients in the non-deficient group were older (60.46 ± 12.86 vs. 56.83 ± 12.86 years, *P* = 0.004), had a higher proportion of males (71.7% vs. 49.6%, *P* < 0.001), and had a longer duration of diabetes (10.0 [4.0–15.0] vs. 7.0 [2.0–12.0] years, *P* = 0.005). No significant difference in BMI was observed between the two groups (*P* = 0.373).

Regarding laboratory parameters, the vitamin D deficient group exhibited significantly higher levels of HbA1c (8.94 ± 1.97 vs. 8.27 ± 1.91, *P* < 0.001), triglycerides (1.88 vs. 1.47 mmol/L, *P* < 0.001), and LDL-C (3.48 ± 1.24 vs. 3.00 ± 1.04 mmol/L, *P* < 0.001). No significant differences were found in albumin, hemoglobin, serum calcium, serum phosphorus, or eGFR between groups (all *P* > 0.05).

For UACR, levels were significantly higher in the vitamin D deficient group (12.13 [3.99–60.34] vs. 7.45 [2.43–31.79] mg/g, *P* = 0.009). Although the prevalence of DR was higher in the deficient group (17.7% vs. 10.3%), the difference did not reach statistical significance (*P* = 0.053).

No significant differences were observed between the two groups in terms of hypertension, ASCVD, or medication use, including metformin, GLP-1 receptor agonists, SGLT2 inhibitors, insulin, other antidiabetic agents, and ACEI/ARB (all *P* > 0.05).

In the expanded cohort of 970 patients with T2DM, serum 25(OH)D levels showed significant seasonal variation, with the lowest mean concentration observed in autumn (17.06 ± 5.17 ng/mL), while levels in winter, spring, and summer were relatively higher and comparable (19.64 ± 5.75, 19.32 ± 5.61, and 19.50 ± 5.06 ng/mL, respectively); detailed results are presented in Supplementary Fig. S1.

**Table 1:** Baseline characteristics of patients with T2DM stratified by serum 25(OH)D levels

Variable	Total (*N* = 518)	25(OH)D < 20 ng/mL (*n* = 372)	25(OH)D ≥ 20 ng/mL (*n* = 146)	*P*-value
**Demographics**				
Age, years	57.84 ± 12.95	56.83 ± 12.86	60.46 ± 12.86	0.004
Male sex, n (%)	289 (55.8)	185 (49.6)	104 (71.7)	< 0.001
Duration of T2DM, years	7.0 (2.0–13.0)	7.0 (2.0–12.0)	10.0 (4.0–15.0)	0.005
BMI, kg/m²	26.43 ± 3.88	26.51 ± 4.05	26.20 ± 3.38	0.373
**Laboratory parameters**				
HbA1c, %	8.75 ± 1.97	8.94 ± 1.97	8.27 ± 1.91	< 0.001
TG, mmol/L	1.77 (1.19–2.64)	1.88 (1.24–2.90)	1.47 (1.10–2.12)	< 0.001
LDL-C, mmol/L	3.34 ± 1.21	3.48 ± 1.24	3.00 ± 1.04	< 0.001
Albumin, g/L	39.82 ± 4.68	39.76 ± 4.96	40.00 ± 3.86	0.554
Hemoglobin, g/L	139.69 ± 19.18	139.17 ± 19.66	141.00 ± 17.88	0.311
Serum calcium, mmol/L	2.06 ± 0.14	2.06 ± 0.15	2.06 ± 0.12	0.888
Serum phosphorus, mmol/L	1.24 ± 0.22	1.24 ± 0.22	1.24 ± 0.23	0.873
eGFR, mL/min/1.73 m²	95.10 ± 20.03	95.61 ± 19.98	93.81 ± 20.16	0.363
UACR, mg/g	10.69 (3.32–49.19)	12.13 (3.99–60.34)	7.45 (2.43–31.79)	0.009
25(OH)D, ng/mL	17.77 (15.19–20.56)	16.25 (14.42–18.09)	22.63 (21.29–25.78)	< 0.001
**Complications**,** n (%)**				
Hypertension	271 (52.3)	189 (50.7)	82 (56.6)	0.269
ASCVD	423 (81.7)	300 (80.4)	123 (84.8)	0.301
DR	81 (15.6)	66 (17.7)	15 (10.3)	0.053
**Medications**,** n (%)**				
Metformin	243 (46.9)	173 (46.4)	70 (48.3)	0.772
GLP-1 RA	26 (5.0)	18 (4.8)	8 (5.5)	0.921
SGLT2i	102 (19.7)	68 (18.2)	34 (23.4)	0.223
Insulin	186 (35.9)	129 (34.6)	57 (39.3)	0.366
Other antidiabetics	150 (29.0)	102 (27.3)	48 (33.1)	0.234
ACEI/ARB	115 (22.2)	77 (20.6)	38 (26.2)	0.211

**Notes**:T2DM, type 2 diabetes mellitus; BMI, body mass index; TG, triglycerides; LDL-C, low-density lipoprotein cholesterol; eGFR, estimated glomerular filtration rate; UACR, urinary albumin-to-creatinine ratio; 25(OH)D, 25-hydroxyvitamin D; ASCVD, atherosclerotic cardiovascular disease; DR, diabetic retinopathy; GLP-1 RA, glucagon-like peptide-1 receptor agonist; SGLT2i, sodium-glucose cotransporter 2 inhibitor; ACEI/ARB, angiotensin-converting enzyme inhibitor/angiotensin receptor blocker

Data are presented as mean ± SD, median (interquartile range), or n (%), as appropriate. P values were calculated using the t test, Mann–Whitney U test, or chi-square test, as appropriate

### Association between serum 25(OH)D and UACR

Unless otherwise specified, all multivariable models were adjusted for age, sex, BMI, diabetes duration, HbA1c, hypertension, eGFR, and use of ACEI/ARB, SGLT2 inhibitors, insulin, and metformin. Both crude and multivariable-adjusted estimates are presented to allow assessment of potential confounding.

#### Linear regression analysis of serum 25(OH)D and ln(UACR)

To address potential bias arising from missing data, multiple imputation was applied in the primary analysis (Table 2). In the univariable linear regression model, serum 25(OH)D levels were inversely associated with ln(UACR) (B = − 0.071, 95% CI: -0.109 to -0.033, P **< 0.001**). This association remained statistically significant after multivariable adjustment for age, sex, BMI, duration of T2DM, HbA1c, hypertension, eGFR, and medication use (B = -0.070, 95% CI: -0.106 to -0.033, *P* < 0.001).

In the fully adjusted model, male sex (B = 0.390, *P* = 0.023), longer duration of T2DM (B = 0.030, *P* = 0.029), higher HbA1c (B = 0.216, *P* < 0.001), and hypertension (B = 0.645, *P* < 0.001) were positively associated with ln(UACR), whereas age (B = − 0.021, *P* = 0.024) and eGFR (B = − 0.036, *P* < 0.001) were inversely associated with ln(UACR). No statistically significant associations were observed for BMI or the use of ACEI/ARB, SGLT2 inhibitors, metformin, or insulin (all *P* > 0.05).

To evaluate the robustness of these findings, a sensitivity analysis was conducted using the complete-case dataset (Supplementary Table S3). The direction and magnitude of the associations were comparable to those observed in the primary imputed analyses. In the crude model, 25(OH)D remained inversely associated with ln(UACR) (B = − 0.064, 95% CI: −0.104 to − 0.024, *P* = 0.002), and this association remained statistically significant after multivariable adjustment (B = − 0.070, 95% CI: −0.108 to − 0.032, *P* < 0.001).

#### Logistic regression analysis of serum 25(OH)D and UACR ≥ 30 mg/g

To further examine the association between serum 25(OH)D and UACR ≥ 30 mg/g, logistic regression analyses were performed in the multiply imputed dataset (Table 3). In the univariable model, serum 25(OH)D was inversely associated with the odds of UACR ≥ 30 mg/g (OR = 0.940, 95% CI: 0.898–0.984, *P* = 0.008). This association remained statistically significant after multivariable adjustment (OR = 0.933, 95% CI: 0.887–0.981, *P* = 0.006).

In the fully adjusted model, male sex (OR = 1.801, *P* = 0.011), higher HbA1c (OR = 1.293, *P* < 0.001), and hypertension (OR = 2.026, *P* = 0.004) were associated with higher odds of UACR ≥ 30 mg/g, whereas higher eGFR was associated with lower odds (OR = 0.958, *P* < 0.001). Age, BMI, duration of T2DM, and the use of ACEI/ARB, SGLT2 inhibitors, insulin, and metformin were not statistically significantly associated with UACR ≥ 30 mg/g (all *P* > 0.05).

Sensitivity analyses based on the complete-case dataset yielded consistent results (Supplementary Table S4). Serum 25(OH)D remained inversely associated with UACR ≥ 30 mg/g in both the crude model (OR = 0.947, 95% CI: 0.904–0.992, *P* = 0.022) and the adjusted model (OR = 0.936, 95% CI: 0.890–0.984, *P* = 0.009).

#### Subgroup and interaction analyses

In the multiply imputed dataset, serum 25(OH)D remained inversely associated with UACR ≥ 30 mg/g after multivariable adjustment (*R* = 0.933, 95% CI: 0.887–0.981, *P* = 0.006). Subgroup and interaction analyses were conducted to evaluate potential effect modification.

A statistically significant interaction was observed between 25(OH)D and age (P for interaction = 0.002) (Table 4). Age was dichotomized at 59 years, corresponding approximately to the median of the study population. The association between 25(OH)D and UACR ≥ 30 mg/g was statistically significant in participants aged < 59 years (OR = 0.855, 95% CI: 0.773–0.947, *P* = 0.003), but not in those aged ≥ 59 years (OR = 0.968, 95% CI: 0.911–1.029, *P* = 0.295).

Consistent patterns were observed using alternative age cut-offs. At the 70-year cut-off, a statistically significant association was observed only in the younger subgroup (OR = 0.928, 95% CI: 0.874–0.986, *P* = 0.016). Similarly, at the 50-year cut-off, the association was statistically significant in participants aged < 50 years (OR = 0.786, 95% CI: 0.653–0.945, *P* = 0.011), but not in those aged ≥ 50 years (OR = 0.953, 95% CI: 0.904–1.004, *P* = 0.073).

No statistically significant interaction was observed for T2DM duration, sex, ACEI/ARB use, or eGFR (Supplementary Table S5).

#### Dose–response relationship between serum 25(OH)D and UACR

To further characterize the dose–response relationship, RCS models were constructed with four knots placed at the 5th, 35th, 65th, and 95th percentiles of serum 25(OH)D (11.87, 16.18, 19.01, and 26.86 ng/mL).

**Fig. 2:**
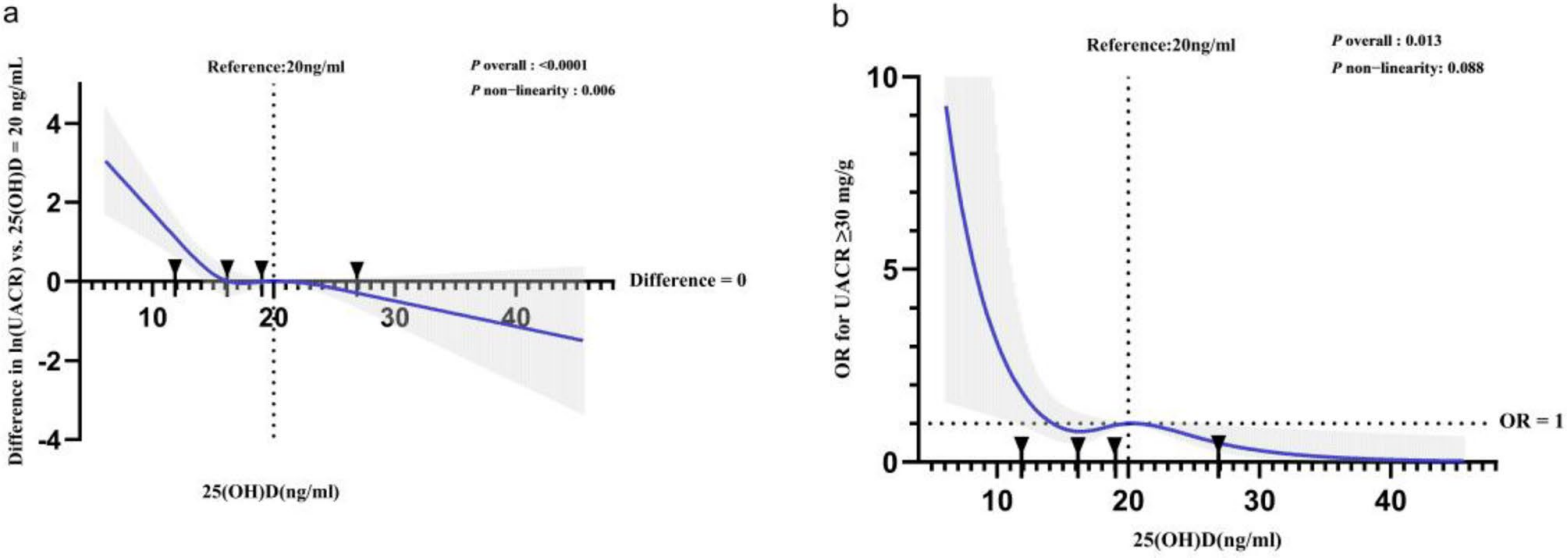
Dose-response relationship of serum 25(OH)D with ln(UACR) and the risk of UACR≥30 mg/g. (**a**) RCS curve for the association between serum 25(OH)D and ln(UACR). The solid line represents the multivariable-adjusted difference in predicted ln(UACR) relative to the reference value (25(OH)D = 20 ng/mL), where UACR is expressed in mg/g and natural log-transformed [ln(UACR)]. The shaded area represents the 95% CIs. The P for overall association was < 0.0001, and the P for non-linearity was 0.006. The reference value was set at 20 ng/mL. The four black triangles on the curve indicate the positions of the knots at the 5th, 35th, 65th, and 95th percentiles (11.87, 16.18, 19.01, and 26.86 ng/mL. (**b**) RCS curve for UACR ≥ 30 mg/g. The solid line represents the multivariable-adjusted odds ratios for UACR ≥ 30 mg/g, and the shaded area represents the 95% CIs. The reference value was set at 20 ng/mL. The P for overall association was 0.013, and the P for non-linearity was 0.088. The four black triangles on the curve indicate the positions of the knots at the 5th, 35th, 65th, and 95th percentiles (11.87, 16.18, 19.01, and 26.86 ng/mL)

As shown in Fig. 2a, serum 25(OH)D concentration was inversely associated with ln(UACR), with evidence of non-linearity (P for overall association < 0.0001; P for non-linearity = 0.006). Using 20 ng/mL as the reference, ln(UACR) values were higher at lower concentrations of 25(OH)D (e.g., 10 ng/mL: β = 1.72, 95% CI: 0.97–2.46), whereas differences in ln(UACR) were smaller at higher concentrations (e.g., 30 ng/mL: β = −0.49, 95% CI: −1.12 to 0.13).

Using the same model specification, serum 25(OH)D concentration was inversely associated with the odds of UACR ≥ 30 mg/g (Fig. 2b). Compared with the reference value of 20 ng/mL, the odds were higher at lower concentrations (e.g., 10 ng/mL: OR = 3.04, 95% CI: 1.15–8.01), and lower at higher concentrations (e.g., 30 ng/mL: OR = 0.30, 95% CI: 0.10–0.90). The overall association was statistically significant (*P* = 0.013), whereas the test for non-linearity was not statistically significant (*P* = 0.088).

**Table 2:** Association between serum 25(OH)D and ln(UACR) in patients with T2DM: results from multiple imputation analysis

Variables	B	SE	95% CI	*P* value
**Model 1 (Crude)**				
25(OH)D (ng/mL)	-0.071	0.019	-0.109 – -0.033	**< 0.001**
**Model 2 (Adjusted)**				
25(OH)D (ng/mL)	-0.070	0.019	-0.106 – -0.033	**< 0.001**
Age (years)	-0.021	0.009	-0.040 – -0.003	**0.024**
Sex (male)	0.390	0.172	0.054–0.726	**0.023**
BMI (kg/m²)	0.014	0.023	-0.031–0.059	0.549
Duration of T2DM (years)	0.030	0.014	0.003–0.057	**0.029**
HbA1c (%)	0.216	0.045	0.129–0.304	**< 0.001**
Hypertension (yes)	0.645	0.186	0.281–1.009	**< 0.001**
eGFR (mL/min/1.73 m²)	-0.036	0.005	-0.047 – -0.026	**< 0.001**
ACEI/ARB (yes)	-0.316	0.222	-0.751–0.120	0.155
SGLT2i (yes)	-0.002	0.213	-0.420–0.415	0.991
Insulin (yes)	0.311	0.207	-0.094–0.716	0.133
Metformin (yes)	0.180	0.169	-0.152–0.512	0.287

**Notes**:Estimates and standard errors were derived from multiple imputation datasets (m = 20) and combined using Rubin’s rules

Model 1: Crude model (unadjusted)

Model 2: Adjusted for age, sex, BMI, duration of T2DM, HbA1c, hypertension, eGFR, and use of ACEI/ARB, SGLT2i, metformin, and insulin

Abbreviations: B, unstandardized regression coefficient; SE, standard error; CI, confidence interval; 25(OH)D, 25-hydroxyvitamin D; ln(UACR), natural logarithm of urinary albumin-to-creatinine ratio; T2DM, type 2 diabetes mellitus; BMI, body mass index; HbA1c, glycated hemoglobin; eGFR, estimated glomerular filtration rate; ACEI/ARB, angiotensin-converting enzyme inhibitors/angiotensin receptor blockers; SGLT2i, sodium-glucose cotransporter-2 inhibitors

**Table 3:** Association between serum 25(OH)D and UACR ≥ 30 mg/g in patients with T2DM: results from multiple imputation analysis

Variables	β (SE)	OR (95% CI)	*P* value
**Model 1 (Crude)**			
25(OH)D (ng/mL)	-0.062 (0.023)	0.940 (0.898–0.984)	**0.008**
**Model 2 (Adjusted)**			
25(OH)D (ng/mL)	-0.070 (0.026)	0.933 (0.887–0.981)	**0.006**
Age (years)	-0.023 (0.013)	0.977 (0.953–1.002)	0.073
Sex (male)	0.588 (0.231)	1.801 (1.145–2.833)	**0.011**
BMI (kg/m²)	0.017 (0.030)	1.018 (0.960–1.079)	0.557
Duration of T2DM	0.022 (0.018)	1.022 (0.987–1.058)	0.214
HbA1c (%)	0.257 (0.060)	1.293 (1.149–1.455)	**< 0.001**
Hypertension (yes)	0.706 (0.244)	2.026 (1.256–3.268)	**0.004**
eGFR (mL/min/1.73 m²)	-0.042 (0.008)	0.958 (0.944–0.973)	**< 0.001**
ACEI/ARB (yes)	-0.307 (0.281)	0.736 (0.424–1.276)	0.275
SGLT2i (yes)	0.042 (0.284)	1.043 (0.598–1.818)	0.883
Insulin (yes)	0.354 (0.265)	1.425 (0.847–2.397)	0.182
Metformin (yes)	0.424 (0.225)	1.528 (0.984–2.372)	0.059

Notes: Estimates were derived from multiple imputation datasets (m = 20) and combined using Rubin’s rules to account for missing covariate data

Model 1: Crude model (unadjusted)

Model 2: Adjusted for age, sex, BMI, duration of T2DM, HbA1c, hypertension, eGFR, and use of ACEI/ARB, SGLT2i, metformin, and insulin

Abbreviations: β, regression coefficient; SE, standard error; OR, odds ratio; CI, confidence interval; 25(OH)D, 25-hydroxyvitamin D; UACR, urinary albumin-to-creatinine ratio; T2DM, type 2 diabetes mellitus; BMI, body mass index; HbA1c, glycated hemoglobin; eGFR, estimated glomerular filtration rate; ACEI/ARB, angiotensin-converting enzyme inhibitors/angiotensin receptor blockers; SGLT2i, sodium-glucose cotransporter-2 inhibitors

**Table 4:** Age-stratified association between serum 25(OH)D and UACR ≥ 30 mg/g in patients with T2DM

Stratification	*n*	β (SE)	OR (95% CI)	*P*-value	*P* for interaction
Total population	518	-0.070 (0.026)	0.933 (0.887–0.981)	**0.006**	
Age					**0.002**
< 59 years	246	-0.156 (0.052)	0.855 (0.773–0.947)	**0.003**	
≥ 59 years	272	-0.032 (0.031)	0.968 (0.911–1.029)	0.295	

Notes: Age was dichotomized at 59 years, approximately the median of the study population. The P for interaction was obtained from the product term of 25(OH)D × age in the multivariable logistic regression model. Both models were adjusted for sex, BMI, duration of T2DM, HbA1c, hypertension, eGFR, and medications (ACEI/ARB, SGLT2i, metformin, and insulin). Results from alternative cut-offs and other interaction terms are presented in Supplementary Table S5

### Association between 25(OH)D and DR

#### Logistic regression analysis of serum 25(OH)D and DR

To evaluate the association between serum 25(OH)D and the presence of DR, logistic regression analyses were performed in the multiply imputed dataset (Table 5). In the univariable model, serum 25(OH)D was inversely associated with the odds of DR (OR = 0.923, 95% CI: 0.869–0.981, *P* = 0.009). This association remained statistically significant after multivariable adjustment for age, sex, BMI, duration of T2DM, HbA1c, hypertension, eGFR, and medication use (OR = 0.932, 95% CI: 0.877–0.991, *P* = 0.024).

In the fully adjusted model, longer duration of T2DM (OR = 1.052, *P* = 0.013) and insulin use (OR = 2.967, *P* < 0.001) were associated with higher odds of DR. Age, sex, BMI, HbA1c, hypertension, eGFR, and the use of ACEI/ARB, SGLT2 inhibitors, and metformin were not statistically significantly associated with DR (all *P* > 0.05).

Sensitivity analyses based on the complete-case dataset yielded consistent results (Supplementary Table S6). The direction and magnitude of the associations were comparable to those observed in the primary imputed analyses. Serum 25(OH)D remained inversely associated with DR in both the crude model (OR = 0.932, 95% CI: 0.876–0.991, *P* = 0.024) and the adjusted model (OR = 0.940, 95% CI: 0.885–0.999, *P* = 0.046).

#### Subgroup and interaction analyses

To assess potential effect modification, subgroup and interaction analyses were performed. No statistically significant interactions were observed between serum 25(OH)D and any of the examined variables for the odds of DR (all P for interaction > 0.05).

Using 59 years (the pre-specified median) as the cut-off, the association between 25(OH)D and DR did not differ significantly between participants aged < 59 years and those aged ≥ 59 years (P for interaction = 0.919; Table 6). Similarly, no statistically significant interaction was observed for T2DM duration, sex, ACEI/ARB use, or eGFR. Alternative cut-offs for age (50 and 70 years) and T2DM duration (5, 7, and 10 years) yielded consistent results without statistically significant interactions (Supplementary Table S7).

#### Dose–response relationship between serum 25(OH)D and DR

To further characterize the dose–response relationship, a RCS model was constructed with four knots placed at the 5th, 35th, 65th, and 95th percentiles of serum 25(OH)D (11.87, 16.18, 19.01, and 26.86 ng/mL).

**Fig. 3:**
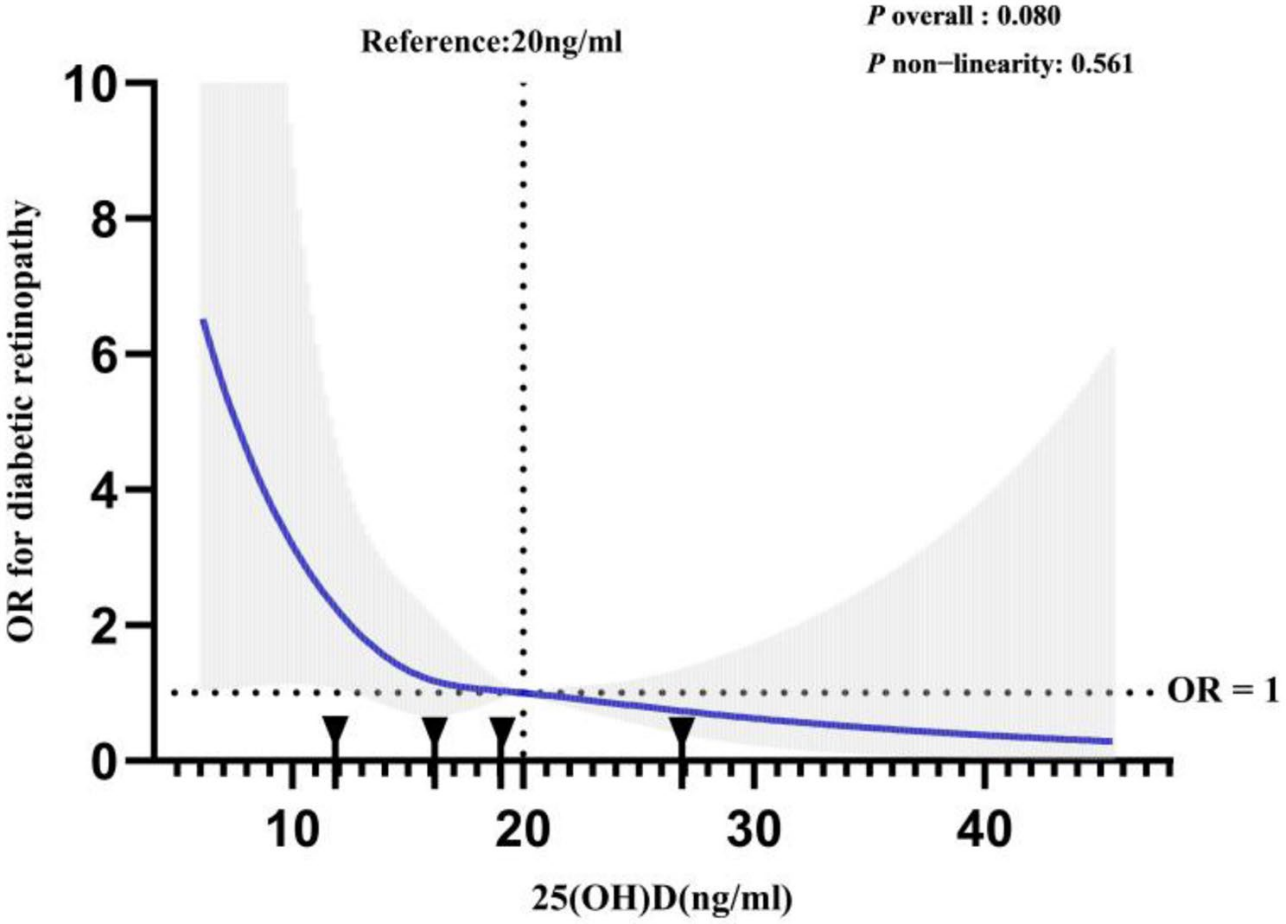
RCS curve for the association between serum 25(OH)D and the risk of DR. The solid line represents multivariable-adjusted odds ratios for DR, relative to the reference value (25(OH)D = 20 ng/mL), and the shaded area represents the 95% confidence intervals. The four black triangles on the curve indicate the positions of the knots at the 5th, 35th, 65th, and 95th percentiles (11.87, 16.18, 19.01, and 26.86 ng/mL). The reference value was set at 20 ng/mL. P for overall association = 0.080; P for non-linearity = 0.561

As shown in Fig. 3, serum 25(OH)D concentration was not statistically significantly associated with the odds of DR (P for overall association = 0.080), and no evidence of non-linearity was observed (P for non-linearity = 0.561). Using 20 ng/mL as the reference value, the estimated odds ratios were numerically higher at lower concentrations and lower at higher concentrations; however, these differences did not reach statistical significance across the observed range.

**Table 5:** Association between serum 25(OH)D and DR in patients with T2DM: results from multiple imputation analysis

Variables	β (SE)	OR (95% CI)	*P* value
**Model 1 (Crude)**			
25(OH)D (ng/mL)	-0.080 (0.031)	0.923 (0.869–0.981)	**0.009**
**Model 2 (Adjusted)**			
25(OH)D (ng/mL)	-0.070 (0.031)	0.932 (0.877–0.991)	**0.024**
Age (years)	-0.001 (0.015)	0.999 (0.969–1.029)	0.933
Sex (male)	-0.509 (0.280)	0.601 (0.348–1.040)	**0.069**
BMI (kg/m²)	0.011 (0.036)	1.011 (0.942–1.085)	0.762
Duration of T2DM	0.051 (0.021)	1.052 (1.011–1.095)	**0.013**
HbA1c (%)	0.102 (0.073)	1.107 (0.959–1.278)	0.164
Hypertension (yes)	-0.185 (0.298)	0.831 (0.464–1.490)	0.535
eGFR (mL/min/1.73 m²)	-0.005 (0.008)	0.995 (0.980–1.010)	0.487
ACEI/ARB (yes)	-0.117 (0.352)	0.889 (0.446–1.773)	0.739
SGLT2i (yes)	-0.136 (0.346)	0.873 (0.443–1.720)	0.694
Insulin (yes)	1.087 (0.318)	2.967 (1.590–5.534)	**< 0.001**
Metformin (yes)	0.181 (0.276)	1.198 (0.697–2.059)	0.512

Notes: Estimates and standard errors were derived from multiple imputation datasets (m = 20) and combined using Rubin’s rules

Model 1: Crude model (unadjusted)

Model 2: Adjusted for age, sex, BMI, duration of T2DM, HbA1c, hypertension, eGFR, and use of ACEI/ARB, SGLT2i, insulin, and metformin

Abbreviations: β, regression coefficient; SE, standard error; OR, odds ratio; CI, confidence interval; 25(OH)D, 25-hydroxyvitamin D; DR, diabetic retinopathy; T2DM, type 2 diabetes mellitus; BMI, body mass index; HbA1c, glycated hemoglobin; eGFR, estimated glomerular filtration rate; ACEI/ARB, angiotensin-converting enzyme inhibitors/angiotensin receptor blockers; SGLT2i, sodium-glucose cotransporter-2 inhibitors

**Table 6:** Age-stratified association between serum 25(OH)D and the risk of DR in patients with T2DM

Stratification	*n*	β (SE)	OR (95% CI)	*P*-value	*P* for interaction
Total population	518	-0.070 (0.031)	0.932 (0.877–0.991)	**0.024**	
Age					0.919
< 59 years	246	-0.101 (0.057)	0.904 (0.809–1.010)	0.074	
≥ 59 years	272	-0.043 (0.039)	0.958 (0.888–1.035)	0.276	

Notes: To maintain methodological consistency, age was dichotomized at 59 years, corresponding to the median of the study population used in the UACR analysis. The P for interaction was obtained from the product term of 25(OH)D × age in the multivariable logistic regression model. Both models were adjusted for sex, BMI, duration of T2DM, HbA1c, hypertension, eGFR, and medications (ACEI/ARB, SGLT2i, metformin, and insulin). Results from alternative cut-offs and other interaction terms are presented in Supplementary Table S6

## Discussion

### Serum 25(OH)D and UACR

In this cross-sectional study of hospitalized patients with T2DM, serum 25(OH)D was inversely associated with UACR after multivariable adjustment and multiple imputation.A significant interaction with age was observed, indicating age-related heterogeneity in the association between serum 25(OH)D and UACR. The inverse association was more evident in younger participants, whereas it was attenuated in older individuals. No significant interaction was observed for diabetes duration, sex, ACEI/ARB use, or eGFR.

This age-related heterogeneity may reflect reduced renal reserve and diminished biological responsiveness with aging [17–20]. However, the study population was predominantly within the A1 range of UACR, so the observed association should be interpreted as variation in a mainly low-UACR cohort rather than evidence directly applicable to more advanced UACR elevations. Prior observational studies that included broader diabetic populations and overt kidney involvement have reported stronger associations [12, 18], which limits the generalizability of the present findings.

### Serum 25(OH)D and DR

Serum 25(OH)D was inversely associated with DR in multivariable logistic regression, but the association was modest. No significant interactions were observed for age, diabetes duration, sex, ACEI/ARB use, or eGFR. In addition, the restricted cubic spline analysis did not show a statistically significant overall association or non-linearity. Taken together, the DR results indicate a statistical association, but not a robust dose–response pattern.

The present DR findings are broadly consistent with prior observational reports linking lower vitamin D status to DR-related phenotypes [14–16]. However, the lack of a significant spline signal indicates that the association should be interpreted cautiously and as a modest statistical relationship rather than a strong concentration-dependent pattern.

### Biological plausibility and interpretation

Vitamin D has been linked to inflammation, oxidative stress, and renin–angiotensin system regulation [6–9], which may be relevant to variation in UACR and DR. Vitamin D has also been discussed in the broader context of glycemic regulation and diabetes-related risk [10–13]. However, the cross-sectional design does not allow temporal ordering or causal inference. Reverse causation is possible. For UACR, urinary loss of vitamin D-binding protein may be associated with lower circulating 25(OH)D. For DR, reduced outdoor activity in patients with visual impairment may lower sunlight exposure and vitamin D synthesis. Residual confounding from diet, activity, sunlight exposure, and supplementation cannot be excluded.

### Seasonal variation and study limitations

Seasonal analysis showed that serum 25(OH)D varied across seasons in T2DM, with the lowest levels in autumn and no significant differences among spring, summer, and winter. This analysis describes the distribution of the exposure rather than the association with UACR or DR, and therefore does not strengthen causal interpretation. Although multiple imputation and complete-case analyses yielded consistent results, the single-center inpatient setting and the predominance of A1-range UACR limit the generalizability of the findings to community-based patients or those with higher UACR levels. In addition, restricting the study population to a single endocrinology inpatient department may have introduced selection bias, as hospitalized patients with T2DM may represent a more severe clinical phenotype and may have lower 25(OH)D concentrations because of reduced physical activity and limited sunlight exposure. The exclusion of patients with eGFR < 30 mL/min/1.73 m² further narrows the spectrum of renal impairment represented. The cutoff of 20 ng/mL was used to define vitamin D deficiency, which is commonly applied in the literature [4, 5], but alternative thresholds exist and may alter classification and interpretation.

### Relationship to intervention evidence

The present observational findings should not be interpreted as evidence that vitamin D supplementation improves microvascular phenotypes. In particular, the VITAL-DKD trial [21], the largest randomized controlled trial of vitamin D supplementation in T2DM to date, did not show a significant effect of vitamin D3 on eGFR decline or urinary albumin excretion over five years. For DR, adequately powered interventional evidence remains limited. Accordingly, the present results should be viewed as evidence of association rather than support for supplementation as a preventive or therapeutic strategy.

## Conclusion

Serum 25(OH)D was inversely associated with UACR and DR in hospitalized patients with T2DM. The association with UACR was more consistent, showed a nonlinear pattern, and appeared to be modified by age, with a stronger inverse association observed in younger participants. The DR association was weaker and did not show a robust dose–response pattern in spline analysis. Because the study was cross-sectional and the cohort was mainly within the A1 range of UACR, these findings should be interpreted as associations with limited clinical generalizability. Prospective studies are needed to clarify whether serum 25(OH)D is a marker or a modifiable correlate of these phenotypes.

## Supplementary Information

Below is the link to the electronic supplementary material.

Supplementary Material 1

Supplementary Material 2

Supplementary Material 3

Supplementary Material 4

Supplementary Material 5

Supplementary Material 6

Supplementary Material 7

Supplementary Material 8

## Data Availability

The datasets generated and/or analyzed during the current study are included in this published article and its supplementary material.
