# Supplementary material for: Association of serum 25-hydroxyvitamin D with urinary albumin-to-creatinine ratio and diabetic retinopathy in hospitalized patients with type 2 diabetes mellitus: a cross-sectional study
Source: BMC Endocr Disord. 2026 May 11;26:194. doi: 10.1186/s12902-026-02307-w (PMC13335294; doi:10.1186/s12902-026-02307-w)
Supplement: Supplementary file 1 — Supplementary Material 1 [file 12902_2026_2307_MOESM1_ESM.docx]

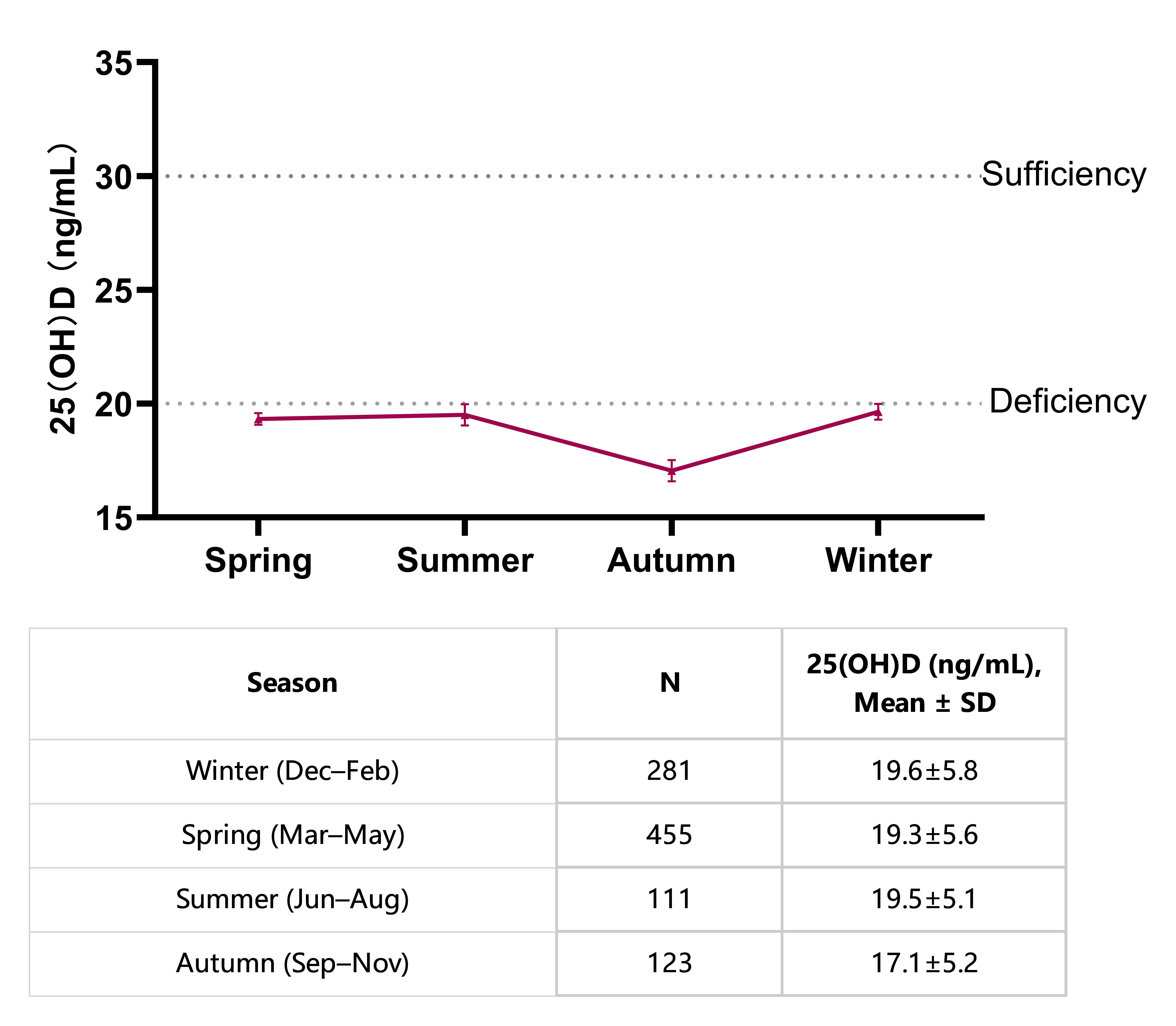


**Fig. S1 Seasonal variation in serum 25(OH)D levels in patients with T2DM.**

Serum 25(OH)D levels across spring, summer, autumn, and winter in patients with T2DM (n = 970) . Data are presented as mean ± standard error of the mean (SEM) for graphical display, and as mean ± D in the embedded table.
