# Supplementary material for: Association of serum 25-hydroxyvitamin D with urinary albumin-to-creatinine ratio and diabetic retinopathy in hospitalized patients with type 2 diabetes mellitus: a cross-sectional study
Source: BMC Endocr Disord. 2026 May 11;26:194. doi: 10.1186/s12902-026-02307-w (PMC13335294; doi:10.1186/s12902-026-02307-w)
Supplement: Supplementary file 4 — Supplementary Material 4 [file 12902_2026_2307_MOESM4_ESM.docx]

Supplementary Table S3. Association between serum 25(OH)D and ln(UACR) in patients with T2DM：results from complete-case dataset

| Variables | B | S.E. | 95% CI | P value |
| --- | --- | --- | --- | --- |
| Model 1 (Crude) |  |  |  |  |
| 25(OH)D (ng/ml) | -0.064 | 0.02 | (-0.104, -0.024) | **0.002** |
| Model 2 (Adjusted) |  |  |  |  |
| 25(OH)D (ng/ml) | -0.07 | 0.019 | (-0.108, -0.032) | **< 0.001** |
| Age (years) | -0.021 | 0.01 | (-0.041, -0.002) | **0.031** |
| Duration of T2DM (years) | 0.037 | 0.014 | (0.009, 0.065) | **0.01** |
| HbA1c (%) | 0.2 | 0.047 | (0.108, 0.291) | **< 0.001** |
| eGFR (mL/min/1.73 m²) | -0.037 | 0.005 | (-0.048, -0.026) | **< 0.001** |
| Hypertension (Yes) | 0.661 | 0.194 | (0.280, 1.042) | **< 0.001** |
| Sex (Male) | 0.375 | 0.179 | (0.023, 0.727) | **0.037** |
| BMI (kg/m²) | 0.024 | 0.024 | (-0.023, 0.071) | 0.316 |
| ACEI/ARB (Yes) | -0.407 | 0.233 | (-0.865, 0.051) | 0.081 |
| Metformin (Yes) | 0.168 | 0.176 | (-0.177, 0.514) | 0.34 |
| Insulin (Yes) | 0.253 | 0.215 | (-0.170, 0.675) | 0.24 |
| SGLT2i (Yes) | -0.004 | 0.222 | (-0.440, 0.432) | 0.986 |

Notes: Estimates and standard errors were calculated using multivariable linear regression analysis based on the complete-case population to assess the robustness of the primary findings.

Model 1: Crude model (unadjusted).

Model 2: Adjusted for age, sex, BMI, T2DM duration, HbA1c, hypertension, eGFR, and use of ACEI/ARB, metformin, insulin, and SGLT2i.

Abbreviations: B, unstandardized regression coefficient; S.E., standard error; CI, confidence interval; 25(OH)D, 25-hydroxyvitamin D; ln(UACR), natural logarithm of urinary albumin-to-creatinine ratio; T2DM, type 2 diabetes mellitus; HbA1c, glycated hemoglobin; eGFR, estimated glomerular filtration rate; BMI, body mass index; ACEI/ARB, angiotensin-converting enzyme inhibitors/angiotensin receptor blockers; SGLT2i, sodium-glucose cotransporter-2 inhibitors.
