# Supplementary material for: Association of serum 25-hydroxyvitamin D with urinary albumin-to-creatinine ratio and diabetic retinopathy in hospitalized patients with type 2 diabetes mellitus: a cross-sectional study
Source: BMC Endocr Disord. 2026 May 11;26:194. doi: 10.1186/s12902-026-02307-w (PMC13335294; doi:10.1186/s12902-026-02307-w)
Supplement: Supplementary file 5 — Supplementary Material 5 [file 12902_2026_2307_MOESM5_ESM.docx]

Supplementary Table S4. Association between serum 25(OH)D and UACR ≥30 mg/g in patients with T2DM: results from complete-case dataset

| Variables | β (SE) | OR (95% CI) | P value |
| --- | --- | --- | --- |
| **Model 1 (Crude)** |  |  |  |
| 25(OH)D (ng/mL) | -0.054 (0.024) | 0.947 (0.904–0.992) | **0.022** |
| **Model 2 (Adjusted)** |  |  |  |
| 25(OH)D (ng/mL) | -0.067 (0.026) | 0.936 (0.890–0.984) | **0.009** |
| Age (years) | -0.025 (0.013) | 0.975 (0.951–1.001) | 0.057 |
| Duration of T2DM (years) | 0.029 (0.018) | 1.029 (0.994–1.067) | 0.108 |
| HbA1c (%) | 0.235 (0.062) | 1.265 (1.120–1.429) | **<0.001** |
| eGFR (mL/min/1.73 m²) | -0.044 (0.008) | 0.957 (0.943–0.972) | **<0.001** |
| Hypertension (Yes) | 0.710 (0.250) | 2.033 (1.244–3.322) | **0.005** |
| Sex (Male) | 0.528 (0.237) | 1.696 (1.066–2.698) | **0.026** |
| BMI (kg/m²) | 0.022 (0.030) | 1.023 (0.963–1.086) | 0.461 |
| ACEI/ARB (Yes) | -0.388 (0.291) | 0.678 (0.384–1.199) | 0.182 |
| Metformin (Yes) | 0.353 (0.228) | 1.423 (0.909–2.226) | 0.123 |
| Insulin (Yes) | 0.273 (0.271) | 1.314 (0.773–2.236) | 0.313 |
| SGLT2i (Yes) | 0.109 (0.288) | 1.115 (0.634–1.961) | 0.706 |

Notes: Estimates (β) and standard errors (SE) were calculated using multivariable logistic regression analysis based on the complete-case population.
Model 1: Crude model (unadjusted).
Model 2: Adjusted for age, sex, BMI, duration of T2DM, HbA1c, hypertension, eGFR, and use of ACEI/ARB, SGLT2i, metformin, and insulin.

Abbreviations: β, regression coefficient; SE, standard error; OR, odds ratio; CI, confidence interval; 25(OH)D, 25-hydroxyvitamin D; UACR, urinary albumin-to-creatinine ratio; T2DM, type 2 diabetes mellitus; BMI, body mass index; HbA1c, glycated hemoglobin; eGFR, estimated glomerular filtration rate; ACEI/ARB, angiotensin-converting enzyme inhibitors/angiotensin receptor blockers; SGLT2i, sodium-glucose cotransporter-2 inhibitors.
