# Supplementary material for: Association of serum 25-hydroxyvitamin D with urinary albumin-to-creatinine ratio and diabetic retinopathy in hospitalized patients with type 2 diabetes mellitus: a cross-sectional study
Source: BMC Endocr Disord. 2026 May 11;26:194. doi: 10.1186/s12902-026-02307-w (PMC13335294; doi:10.1186/s12902-026-02307-w)
Supplement: Supplementary file 6 — Supplementary Material 6 [file 12902_2026_2307_MOESM6_ESM.docx]

Supplementary Table S5a.Subgroup and interaction analyses of the association between serum 25(OH)D and UACR ≥30 mg/g in patients with T2DM

| Subgroups / Factors | n | β (SE) | OR (95% CI) | P-value | P for interaction |
| --- | --- | --- | --- | --- | --- |
| I**nteraction Terms** |  |  |  |  |  |
| 25(OH)D × Age | - | 0.007 (0.002) | - | - | **0.002** |
| 25(OH)D × Duration of T2DM | - | 0.001(0.003) | - | - | 0.607 |
| 25(OH)D × Male sex | - | -0.010 (0.052) | - | - | 0.843 |
| 25(OH)D × ACEI/ARB use | - | -0.047 (0.061) | - | - | 0.442 |
| 25(OH)D × eGFR | - | -0.001 (0.001) | - | - | 0.331 |
| **Alternative Age Cut-offs** |  |  |  |  |  |
| Cutoff: 50 years |  |  |  |  |  |
| < 50 years | 127 | -0.241 (0.094) | 0.786 (0.653–0.945) | **0.011** |  |
| ≥ 50 years | 391 | -0.048 (0.027) | 0.953 (0.904–1.004) | 0.073 |  |
| Cutoff: 70 years |  |  |  |  |  |
| < 70 years | 419 | -0.074 (0.031) | 0.928 (0.874–0.986) | **0.016** |  |
| ≥ 70 years | 99 | -0.065 (0.056) | 0.937 (0.841–1.045) | 0.245 |  |
| T2DM Duration Stratification |  |  |  |  |  |
| Cutoff: 5 years |  |  |  |  |  |
| < 5 years | 191 | -0.159 (0.067) | 0.853 (0.747–0.973) | **0.018** |  |
| ≥ 5 years | 327 | -0.055 (0.027) | 0.947 (0.897–0.999) | **0.047** |  |
| Cutoff: 7 years |  |  |  |  |  |
| < 7 years | 235 | -0.104 (0.053) | 0.901 (0.813–0.999) | **0.048** |  |
| ≥ 7 years | 283 | -0.056 (0.029) | 0.946 (0.893–1.002) | 0.059 |  |
| Cutoff: 10 years |  |  |  |  |  |
| < 10 years | 286 | -0.105 (0.047) | 0.900 (0.822–0.986) | **0.024** |  |
| ≥ 10 years | 232 | -0.048 (0.031) | 0.953 (0.897–1.013) | 0.120 |  |

**Notes:** Estimates were derived from multiple imputation datasets (m=20) and combined using Rubin’s rules. All subgroup models were adjusted for the same covariates used in the primary multivariable model , except for the variable used as a stratifying factor. Interaction terms (25(OH)D × Factor) were tested using multivariable logistic regression.

Abbreviations: OR, odds ratio; CI, confidence interval; 25(OH)D, 25-hydroxyvitamin D; UACR, urinary albumin-to-creatinine ratio; T2DM, type 2 diabetes mellitus; eGFR, estimated glomerular filtration rate; ACEI/ARB, angiotensin-converting enzyme inhibitors/angiotensin receptor blockers.

Supplementary Table S5b. Full multivariable logistic regression model evaluating the interaction between serum 25(OH)D and age on increased urinary albumin excretion (UACR ≥30 mg/g).

| Variables | β (SE) | OR (95% CI) | P value |
| --- | --- | --- | --- |
| **Exposure and Interaction** |  |  |  |
| 25(OH)D (ng/mL) | -0.104 (0.029) | 0.902 (0.851–0.955) | **<0.001** |
| Age (years) | -0.012 (0.013) | 0.988 (0.962–1.014) | 0.370 |
| 25(OH)D × Age | 0.007 (0.002) | 1.007 (1.003–1.012) | **0.002** |
| **Covariates** |  |  |  |
| Sex (male) | 0.520 (0.232) | 1.683 (1.068–2.652) | **0.025** |
| BMI (kg/m²) | 0.012 (0.031) | 1.012 (0.953–1.075) | 0.688 |
| Hypertension (yes) | 0.670 (0.250) | 1.955 (1.198–3.190) | **0.007** |
| HbA1c (%) | 0.246 (0.063) | 1.279 (1.132–1.446) | **<0.001** |
| eGFR (mL/min/1.73 m²) | -0.040 (0.008) | 0.960 (0.946–0.975) | **<0.001** |
| Duration of T2DM (years) | 0.023 (0.018) | 1.023 (0.988–1.059) | 0.199 |
| **Medication Use** |  |  |  |
| ACEI/ARB (yes) | -0.324 (0.286) | 0.724 (0.413–1.268) | 0.259 |
| SGLT2i (yes) | 0.063 (0.290) | 1.065 (0.603–1.881) | 0.827 |
| Insulin (yes) | 0.321 (0.269) | 1.378 (0.813–2.336) | 0.234 |
| Metformin (yes) | 0.421 (0.228) | 1.524 (0.975–2.382) | 0.065 |

Notes: Estimates and standard errors were derived from multiple imputation datasets (m=20) and combined using Rubin’s rules. The outcome (UACR ≥30 mg/g) was analyzed as a binary variable using multivariable logistic regression.

Abbreviations: β, regression coefficient; SE, standard error; OR, odds ratio; CI, confidence interval; 25(OH)D, 25-hydroxyvitamin D; UACR, urinary albumin-to-creatinine ratio; T2DM, type 2 diabetes mellitus; BMI, body mass index; HbA1c, glycated hemoglobin; eGFR, estimated glomerular filtration rate; ACEI/ARB, angiotensin-converting enzyme inhibitors/angiotensin receptor blockers; SGLT2i, sodium-glucose cotransporter-2 inhibitors.
