# Supplementary material for: Association of serum 25-hydroxyvitamin D with urinary albumin-to-creatinine ratio and diabetic retinopathy in hospitalized patients with type 2 diabetes mellitus: a cross-sectional study
Source: BMC Endocr Disord. 2026 May 11;26:194. doi: 10.1186/s12902-026-02307-w (PMC13335294; doi:10.1186/s12902-026-02307-w)
Supplement: Supplementary file 7 — Supplementary Material 7 [file 12902_2026_2307_MOESM7_ESM.docx]

Supplementary Table S6.Association between serum 25(OH)D and DR in patients with T2DM: results from complete-case dataset

| Variables | β (SE) | OR (95% CI) | P value |
| --- | --- | --- | --- |
| **Model 1 (Crude)** |  |  |  |
| 25(OH)D (ng/mL) | -0.071 (0.031) | 0.932 (0.876–0.991) | **0.024** |
| **Model 2 (Adjusted)** |  |  |  |
| 25(OH)D (ng/mL) | -0.062 (0.031) | 0.940 (0.885–0.999) | **0.046** |
| Age (years) | -0.005 (0.016) | 0.995 (0.965–1.026) | 0.732 |
| Duration of T2DM (years) | 0.053 (0.021) | 1.055 (1.012–1.099) | **0.011** |
| HbA1c (%) | 0.100 (0.075) | 1.105 (0.954–1.279) | 0.183 |
| eGFR (mL/min/1.73 m²) | -0.007 (0.008) | 0.993 (0.978–1.009) | 0.393 |
| Hypertension (Yes) | -0.189 (0.301) | 0.827 (0.458–1.493) | 0.529 |
| Sex (Male) | -0.576 (0.283) | 0.562 (0.323–0.979) | **0.042** |
| BMI (kg/m²) | 0.015 (0.037) | 1.015 (0.945–1.091) | 0.678 |
| ACEI/ARB (Yes) | -0.199 (0.362) | 0.819 (0.403–1.666) | 0.582 |
| Metformin (Yes) | 0.181 (0.278) | 1.198 (0.695–2.064) | 0.515 |
| Insulin (Yes) | 1.031 (0.322) | 2.804 (1.491–5.272) | 0.001 |
| SGLT2i (Yes) | -0.137 (0.348) | 0.872 (0.441–1.726) | 0.694 |

Notes: Estimates and standard errors were calculated using multivariable logistic regression analysis based on the complete-case population.
Model 1: Crude model (unadjusted).
Model 2: Adjusted for age, sex, BMI, duration of T2DM, HbA1c, hypertension, eGFR, and use of ACEI/ARB, SGLT2i, metformin, and insulin.

Abbreviations: β, regression coefficient; SE, standard error; OR, odds ratio; CI, confidence interval; 25(OH)D, 25-hydroxyvitamin D; UACR, urinary albumin-to-creatinine ratio; T2DM, type 2 diabetes mellitus; BMI, body mass index; HbA1c, glycated hemoglobin; eGFR, estimated glomerular filtration rate; ACEI/ARB, angiotensin-converting enzyme inhibitors/angiotensin receptor blockers; SGLT2i, sodium-glucose cotransporter-2 inhibitors.
