# Supplementary material for: Association of serum 25-hydroxyvitamin D with urinary albumin-to-creatinine ratio and diabetic retinopathy in hospitalized patients with type 2 diabetes mellitus: a cross-sectional study
Source: BMC Endocr Disord. 2026 May 11;26:194. doi: 10.1186/s12902-026-02307-w (PMC13335294; doi:10.1186/s12902-026-02307-w)
Supplement: Supplementary file 8 — Supplementary Material 8 [file 12902_2026_2307_MOESM8_ESM.docx]

Supplementary Table S7. Subgroup and interaction analyses of the association between serum 25(OH)D and DR in patients with T2DM

| **Subgroups / Factors** | **n** | **β (SE)** | **OR (95% CI)** | **P-value** | **P for interaction** |
| --- | --- | --- | --- | --- | --- |
| Interaction Terms |  |  |  |  |  |
| 25(OH)D × Age (Centered) | - | -0.000 (0.015) | - | - | 0.919 |
| 25(OH)D × T2DM Duration | - | 0.000 (0.003) | - | - | 0.991 |
| 25(OH)D × Male sex | - | -0.036 (0.061) | - | - | 0.560 |
| 25(OH)D × ACEI/ARB use | - | -0.065 (0.076) | - | - | 0.393 |
| 25(OH)D × eGFR | - | 0.001 (0.001) | - | - | 0.583 |
| Alternative Age Cut-offs |  |  |  |  |  |
| Cutoff: 50 years |  |  |  |  |  |
| < 50 years | 127 | -0.059 (0.118) | 0.943 (0.748–1.188) | 0.617 |  |
| ≥ 50 years | 391 | -0.068 (0.033) | 0.934 (0.876–0.996) | **0.038** |  |
| Cutoff: 70 years |  |  |  |  |  |
| < 70 years | 419 | -0.070 (0.036) | 0.932 (0.869–1.000) | 0.050 |  |
| ≥ 70 years | 99 | -0.085 (0.075) | 0.918 (0.792–1.064) | 0.257 |  |
| T2DM Duration Stratification |  |  |  |  |  |
| Cutoff: 5 years |  |  |  |  |  |
| < 5 years | 191 | -0.067 (0.114) | 0.935 (0.748–1.169) | 0.558 |  |
| ≥ 5 years | 327 | -0.061 (0.032) | 0.941 (0.883–1.003) | 0.061 |  |
| Cutoff: 7 years |  |  |  |  |  |
| < 7 years | 235 | -0.075 (0.074) | 0.928 (0.803–1.072) | 0.308 |  |
| ≥ 7 years | 283 | -0.071 (0.035) | 0.931 (0.869–0.998) | **0.042** |  |
| Cutoff: 10 years |  |  |  |  |  |
| < 10 years | 286 | -0.089 (0.063) | 0.915 (0.809–1.034) | 0.155 |  |
| ≥ 10 years | 232 | -0.069 (0.038) | 0.933 (0.866–1.005) | 0.069 |  |

**Notes:** Estimates were derived from multiple imputation datasets (m=20) and combined using Rubin’s rules. All subgroup models were adjusted for the same covariates used in the primary multivariable model , except for the variable used as a stratifying factor. Interaction terms (25(OH)D × Factor) were tested using multivariable logistic regression.

Abbreviations: OR, odds ratio; CI, confidence interval; 25(OH)D, 25-hydroxyvitamin D; UACR, urinary albumin-to-creatinine ratio; T2DM, type 2 diabetes mellitus; eGFR, estimated glomerular filtration rate; ACEI/ARB, angiotensin-converting enzyme inhibitors/angiotensin receptor blockers.
